# Evaluating α-galactosylceramide as an adjuvant for live attenuated influenza vaccines in pigs

**DOI:** 10.1186/s44149-022-00051-x

**Published:** 2022-08-01

**Authors:** Bianca L. Artiaga, Igor Morozov, Russell Ransburgh, Taeyong Kwon, Velmurugan Balaraman, Sabarish V. Indran, Darling Melany De Carvalho Madrid, Weihong Gu, Jamie Henningson, Wenjun Ma, Jürgen A. Richt, John P. Driver

**Affiliations:** 1 Department of Diagnostic Medicine & Pathobiology, College of Veterinary Medicine, Kansas State University, Manhattan, KS 66506 USA; 2 Division of Animal Sciences, University of Missouri, Columbia, MO 65211 USA; 3 Department of Animal Sciences, University of Florida, Gainesville, FL 32611 USA

**Keywords:** Natural killer T cell, Influenza A virus, Vaccine, Live attenuated influenza virus, Adjuvant, α-Galactosylceramide, Swine

## Abstract

Natural killer T (NKT) cells activated with the glycolipid ligand α-galactosylceramide (α-GalCer) stimulate a wide variety of immune cells that enhance vaccine-mediated immune responses. Several studies have used this approach to adjuvant inactivated and subunit influenza A virus (IAV) vaccines, including to enhance cross-protective influenza immunity. However, less is known about whether α-GalCer can enhance live attenuated influenza virus (LAIV) vaccines, which usually induce superior heterologous and heterosubtypic immunity compared to non-replicating influenza vaccines. The current study used the swine influenza challenge model to assess whether α-GalCer can enhance cross-protective immune responses elicited by a recombinant H3N2 LAIV vaccine (TX98ΔNS1) encoding a truncated NS1 protein. In one study, weaning pigs were administered the H3N2 TX98ΔNS1 LAIV vaccine with 0, 10, 50, and 100 μg/kg doses of α-GalCer, and subsequently challenged with a heterologous H3N2 virus. All treatment groups were protected from infection. However, the addition of α-GalCer appeared to suppress nasal shedding of the LAIV vaccine. In another experiment, pigs vaccinated with the H3N2 LAIV, with or without 50 μg/kg of α-GalCer, were challenged with the heterosubtypic pandemic H1N1 virus. Pigs vaccinated with the LAIV alone generated cross-reactive humoral and cellular responses which blocked virus replication in the airways, and significantly decreased virus shedding. On the other hand, combining the vaccine with α-GalCer reduced cross-protective cellular and antibody responses, and resulted in higher virus titers in respiratory tissues. These findings suggest that: (i) high doses of α-GalCer impair the replication and nasal shedding of the LAIV vaccine; and (ii) α-GalCer might interfere with heterosubtypic cross-protective immune responses. This research raise concerns that should be considered before trying to use NKT cell agonists as a possible adjuvant approach for LAIV vaccines.

**Supplementary Information:**

The online version contains supplementary material available at 10.1186/s44149-022-00051-x.

## Introduction

Influenza A viruses (IAVs) are important pathogens for human and animal health (Ito et al. 1998; Ma et al. 2009; Tong et al. 2012; Long et al. 2019). Vaccination is a critical component of IAV control for humans as well as IAV-susceptible livestock species, such as poultry and swine. In the United States, three different vaccine formats are available for humans: (i) injectable tri- or quadrivalent preparations of inactivated influenza virus vaccine (whole-virus, split-virus or subunit) (Barberis et al. 2016; Gouma et al. 2020); (ii) injectable recombinant hemagglutinin (HA) vaccines (L.P. Yang 2013); and (iii) live attenuated influenza virus (LAIV) vaccines administered by an intranasal spray (Maassab 1967; Roubidoux and Schultz-Cherry 2021; USFDA 2018). Inactivated vaccines are relatively simple and economical to produce. However, they provide little cross-protection against heterologous and heterosubtypic IAV strains, and a mismatch of the circulating IAV with the vaccine strains often results in low vaccine efficacy (Flannery et al. 2019; Lewnard and Cobey 2018). LAIV vaccines deliver improved cross-protection against heterologous and heterosubtypic virus strains due to their induction of cross-reactive antibodies and T cells that recognize conserved internal components of influenza viruses (Beyer et al. 2002; Hoft et al. 2011). Nevertheless, the cold-adapted LAIV vaccine approved for humans by the United States Food and Drug Administration, must be reformulated regularly, and unexpectedly poor mismatching of the vaccine with circulating virus strains can reduce the efficacy to below 50 percent (Caspard et al. 2017). Therefore, there remains a critical need to make LAIV vaccines more efficacious by inducing long-term heterosubtypic immunity.

The use of adjuvants can significantly improve the cross-protective heterologous immunity afforded by IAV vaccines. However, conventional adjuvants seldom improve cross-protective immunity against heterosubtypic IAV strains (Tricco et al. 2013; Gouma et al. 2020). Moreover, they are not usually recommended for mucosal vaccines due to physical and chemical barriers that impede adjuvant absorption and because mucosal surfaces preferentially induce tolerance (Tregoning et al. 2018). Furthermore, they appear to increase the risk of Bell’s palsy, which is believed to be caused by inflammation of the craniofacial nerves (Mutsch et al. 2004). Several strategies have been explored to overcome these obstacles, including the use of α-galactosylceramide (α-GalCer), a glycolipid molecule which potently activates invariant natural killer T (NKT) cells. These cells are a subset of T lymphocytes that recognize glycolipid antigens bound to the CD1d molecule and can stimulate a diverse range of innate and adaptive immune functions, including immune reactions in the pulmonary tract (Bendelac et al. 2007; Cerundolo et al. 2009). Numerous mouse studies have shown that activation with α-GalCer, or derivatives of this molecule, stimulates NKT cells to generate CD4^+^ T helper-like immune responses against a wide variety of co-delivered antigens (Van Kaer et al. 2011; Brennan et al. 2013; Li et al. 2010; Sullivan et al. 2010). Moreover, these responses appear to avoid the type of neuronal inflammation associated with other classes of mucosal adjuvant (Youn et al. 2007). A variety of whole inactivated IAV virus and peptide based IAV vaccines have been adjuvanted with α-GalCer derivatives (Ko et al. 2005; Youn et al. 2007; Kamijuku et al. 2008). Kopecky-Bromberg et al. (2009) have demonstrated that this approach can also improve the efficacy of LAIVs in BALB/c mice intranasally vaccinated with the α-GalCer derivative alpha-C-galactosylceramide (α-C-GalCer); mice administered a LAIV vaccine encoding a truncated NS1 protein applied with α-GalCer had reduced morbidity and mortality compared to mice which received the vaccine alone (Kopecky-Bromberg et al. 2009). Despite these promising results, it remains unclear whether α-GalCer-mediated NKT cell stimulation presents a viable approach for enhancing human LAIV vaccines as mice are not natural hosts of IAVs and murine NKT cell frequency and tissue distribution differs substantially from humans. Furthermore, high doses of α-GalCer have been reported to reduce the replication of LAIV vaccines, which can compromise immune protection (Kopecky-Bromberg et al. 2009; Artiaga et al. 2016b).

Hence, the goal of the current study was to investigate the potential of α-GalCer as a LAIV vaccine adjuvant using the pig influenza challenge model. Swine are well suited for this purpose as (i) they are natural hosts for the same IAVs as humans, (ii) they mirror the clinical signs seen in humans, and (iii) they resemble human anatomy and pathogenesis more closely than mice (Starbæk et al. 2018). Additionally, (iv) pigs express NKT cells with similar frequencies and tissue distribution compared to humans (Artiaga et al. 2014; Yang et al. 2017). Overall, we found that adjuvanting a recombinant H3N2 LAIV vaccine with α-GalCer paradoxically compromises the cross-protective immunity usually afforded by this vaccine against a heterosubtypic H1N1 virus challenge. This outcome raises a cautionary note about using this approach for adjuvanting human and swine LAIV vaccines.

## Results

### Response of LAIV-vaccinated pigs to different α-GalCer doses

High doses of α-GalCer have been shown to reduce the efficacy of LAIV vaccines, probably by stimulating immune responses that inhibit virus replication (Kopecky-Bromberg et al. 2009). Thus, we conducted a swine influenza vaccination-challenge experiment (Experiment 1) to identify α-GalCer doses that avoid inhibiting vaccine virus growth. Pigs were intranasally (i.n.) vaccinated with an H3N2 A/Swine/Texas/4199-2/1998 (TX98) IAV encoding a truncated NS1 protein (TX98ΔNS1) (Solórzano et al. 2005), in combination with 0 (vehicle only), 10, 50 or 100 μg/kg of α-GalCer (Table 1, Additional Fig. 1a). An additional control group was sham vaccinated. All pigs were challenged at 28 days post vaccination (d.p.v.) with a heterologous H3N2 A/Swine/Colorado/23619/1999 (CO99) virus and euthanized 5 days post infection (d.p.i.). No adverse reaction was observed in any of the vaccinated and α-GalCer-treated animals throughout the vaccination phase of 28 days.

**Table 1:** Experiment 1 setup

Group	Experimental group	Vaccine	α-GalCer (μg/kg)	Challenge virus^c^	***N***
1	Mock – CO99	Vehicle^a^	Vehicle^b^	H3N2 CO99	3
2	TX98ΔNS1 + 0μg αGC – CO99	TX98ΔNS1	Vehicle	H3N2 CO99	2
3	TX98ΔNS1 + 10μg αGC – CO99	TX98ΔNS1	10	H3N2 CO99	3
4	TX98ΔNS1 + 50μg αGC – CO99	TX98ΔNS1	50	H3N2 CO99	3
5	TX98ΔNS1 + 100μg αGC – CO99	TX98ΔNS1	100	H3N2 CO99	3

^a^Virus-free Dulbecco's Modified Eagle's Medium (DMEM)

^b^50 μL/kg of dimethyl sulfoxide (DMSO) (the volume used to dissolve the 100 μg/kg dose of α-GalCer used in group 5)

^c^1 x 10^6^ TCID_50_ H3N2 A/Swine/Colorado/23619/1999 administered intratracheally (i.t.) in 2 mL of DMEM

**Fig. 1:**
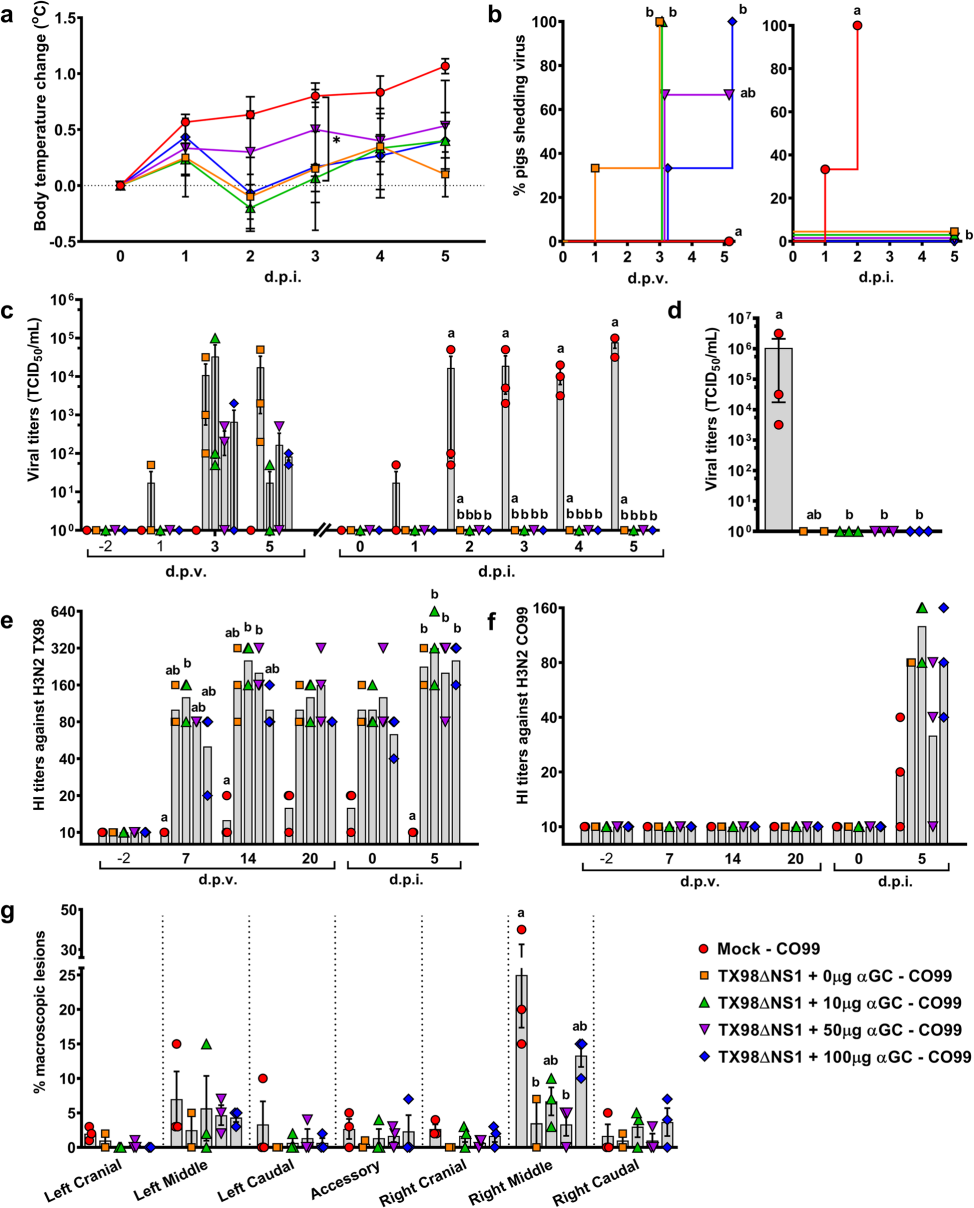
Results of Experiment 1. **a** Change in body temperature during the challenge period based on body temperature at 0 d.p.i.. **b** Percentage of pigs positive for virus shedding in nasal swabs collected at -2, 1, 3, and 5 d.p.v. and 0 to 5 d.p.i. **c** Viral titers in nasal swabs collected after vaccination and challenge. **d** Viral titers in BALF collected at 5 d.p.i.. (**e**, **f**) Geometric mean of serum HI antibody titers against H3N2 TX98 (**e**) and H3N2 CO99 (**f**) collected at -2, 7, 14, and 20 d.p.v. and 0 and 5 d.p.i.. (**g**) Percentage of each lung lobe presenting macroscopic lesions. Differences between treatment groups were determined by Tukey’s (**a**, **g**) or Dunn’s (**c**-**f**) multiple comparisons tests. Survival curves were compared using the Mantel-Cox log-rank test (**b**). A statistically significant difference between two groups is indicated by a star (**a**) or different letters (**b**-**g**). Data are represented as mean ± SEM (**a**-**d**, **g**) or geometric mean (**e**, **f**). Symbols represent treatment groups (**a**, **b**) or individual pigs (**c**-**g**)

After challenge with the heterologous H3N2 CO99 virus, the unvaccinated pig group had higher average body temperatures compared to the vaccinated pigs throughout the 5-day challenge period (Fig. 1a). Although not significant, the incidence of LAIV shedding was delayed in the groups that received 50 or 100 μg/kg of α-GalCer compared to the 0 and 10 μg/kg doses (Fig. 1b). Furthermore, the 50 and 100 μg/kg doses of α-GalCer reduced TX98ΔNS1 virus titers in nasal swabs by ~1-2 log at 3 and 5 d.p.v. compared to pigs that were vaccinated without α-GalCer (Fig. 1c). The 10 μg/kg dose of α-GalCer also reduced virus shedding, but only at 5 d.p.v.. During the challenge period, no virus was detected in nasal swabs or bronchioalveolar lavage fluid (BALF) of any of the vaccinated pigs, regardless of the α-GalCer dose (Fig. 1b-d).

Vaccination with the LAIV induced high H3N2 TX98-specific hemagglutination inhibition (HI) titers in sera regardless of α-GalCer dosage (Fig. 1e). Challenging the vaccinated pigs with CO99 boosted TX98-specific HI titers (Fig. 1e). The H3N2 CO99 challenge also induced modest CO99-specific HI titers at 5 d.p.i. that were similar between vaccinated and unvaccinated pigs (Fig. 1f).

Infection with H3N2 CO99 caused mild lung pathology that mostly affected the right middle lung lobe. Sham-vaccinated pigs had the highest level of macroscopic lesions (Fig. 1g). None of the α-GalCer treatments demonstrated significantly reduced lung pathology compared to pigs that received the LAIV vaccine alone. In fact, lung pathology scores tended to be higher in pigs administered 100 μg/kg α-GalCer compared to the other vaccinated groups.

Collectively, these results demonstrate that the LAIV vaccine protected pigs against infection with the heterologous CO99 virus. They also show that α-GalCer administration did not compromise the ability of the vaccine to clear the challenge virus, despite reducing LAIV levels in nasal swabs. Pigs vaccinated with 100 μg/kg of α-GalCer had the the highest lung pathology scores among the vaccinated groups. Hence, we selected the 50 μg/kg dose to test the adjuvant potential of α-GalCer for enhancing LAIV vaccine protection against a heterosubtypic IAV virus challenge in our second experiment.

### Response of LAIV-vaccinated pigs with or without α-GalCer to heterosubtypic challenge

#### Clinical signs and immunology

**Fig. 2:**
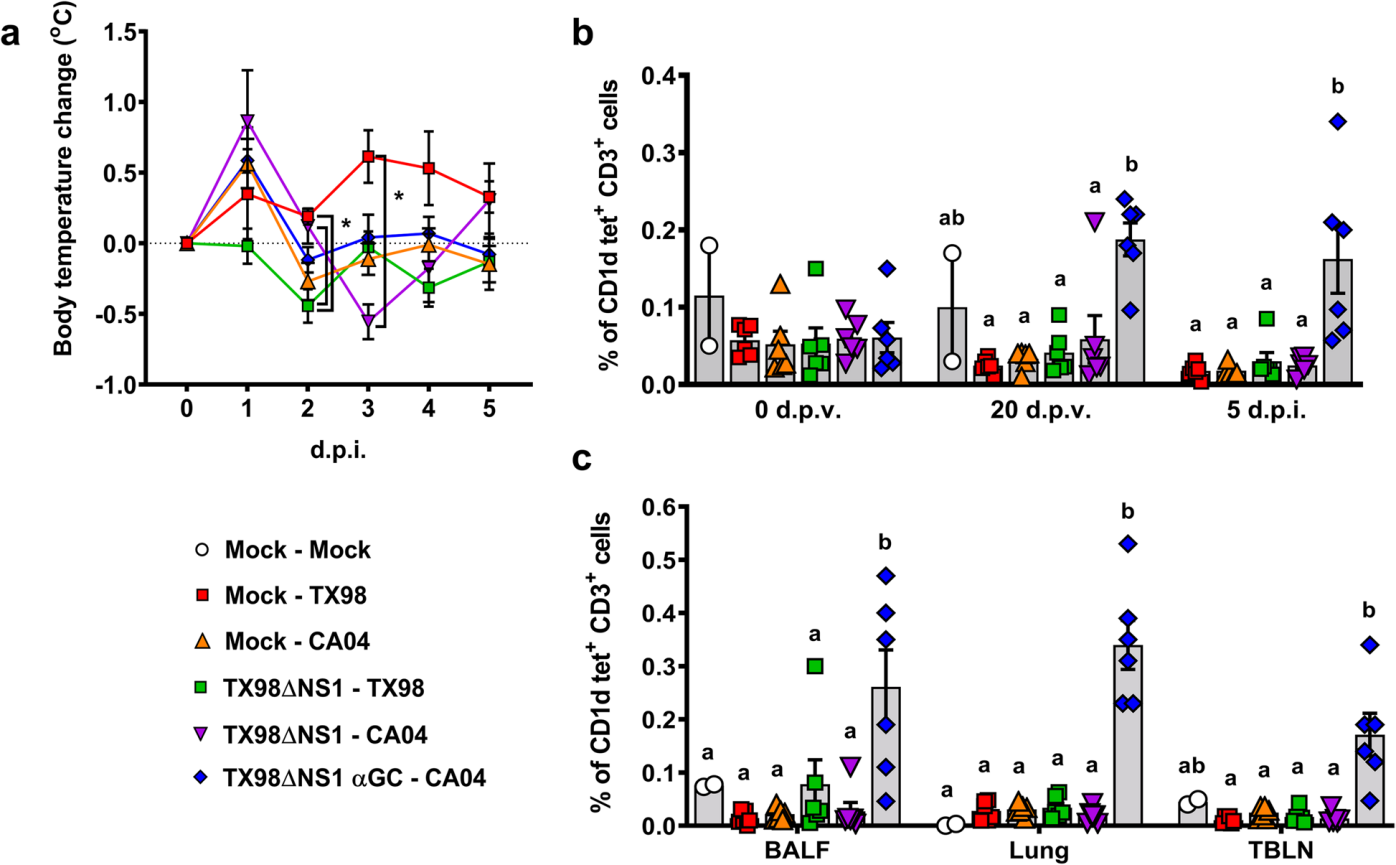
Body temperature and NKT cell frequencies. **a** Change in body temperature during the challenge period was based on the average of the -1 and 0 d.p.i. body temperatures. **b** NKT cells as a proportion of peripheral blood CD3^+^ lymphocytes at 0 and 20 d.p.v. and 5 d.p.i.. **c** NKT cells as a proportion of CD3^+^ lymphocytes in BALF, lung tissue, and TBLN at 5 d.p.i.. Differences between treatment groups were determined by Tukey’s multiple comparisons test. A statistically significant difference between two groups is indicated by a star (**a**) or different letters (**b**, **c**). Data are represented as mean ± SEM. Symbols represent treatment groups (**a**) or individual pigs (**b**, **c**)

In Experiment 2, pigs were vaccinated with H3N2 LAIV TX98ΔNS1, either alone or in combination with 50 μg/kg of α-GalCer and challenged 21 days later with either the homologous wild-type TX98 virus or the heterosubtypic pandemic H1N1 A/California/04/2009 (CA04) virus (Table 2; Additional Fig. 1b). No adverse reaction was observed in any of the vaccinated and α-GalCer-treated animals throughout the vaccination phase of 21 days. After challenge, body temperature was elevated in all three CA04-infected groups at 1 d.p.i and in the unvaccinated pigs infected with TX98 at 3 d.p.i. (Fig. 2a). TX98ΔNS1 vaccinated pigs challenged with the homologous H3N2 TX98 virus did not have pyrexia at any of the timepoints tested.

**Table 2:** Experiment 2 setup

Group	Experimental group	Vaccine	α-GalCer (μg/kg)	Challenge virus^**c**^	***N***
1	Mock – Mock	Vehicle^a^	Vehicle^b^	-	2
2	Mock – TX98	Vehicle	Vehicle	H3N2 TX98	6
3	Mock – CA04	Vehicle	Vehicle	H1N1 CA04	6
4	TX98ΔNS1 – TX98	TX98ΔNS1	Vehicle	H3N2 TX98	6
5	TX98ΔNS1 – CA04	TX98ΔNS1	Vehicle	H1N1 CA04	6
6	TX98ΔNS1 αGC – CA04	TX98ΔNS1	50	H1N1 CA04	6

^a^Virus-free DMEM

^b^25 μL/kg of DMSO (the volume used to dissolve the 50 μg/kg dose of α-GalCer used in group 6)

^c^1 x 10^6^ TCID_50_ H3N2 A/Swine/Texas/4199-2/1998 or H1N1 A/California/04/2009 administered i.t. in 2 mL of DMEM

Flow cytometry was used to compare the frequency of leukocyte populations within blood, BALF, lung tissue, and tracheobronchial lymph node (TBLN) among the different treatment groups in Experiment 2. Pigs that received α-GalCer had higher frequencies of NKT cells in peripheral blood at 20 d.p.v. and 5 d.p.i., and in BALF, lung tissue, and TBLN at 5 d.p.i. compared to the other treatment groups (Fig. 2b and c). However, no differences were detected in the frequency of other types of T cell subsets, natural killer (NK) cells, monocytes, macrophages, dendritic cells, or granulocytes, among the different treatment groups (Additional Figure 2).

#### Serology

**Fig. 3:**
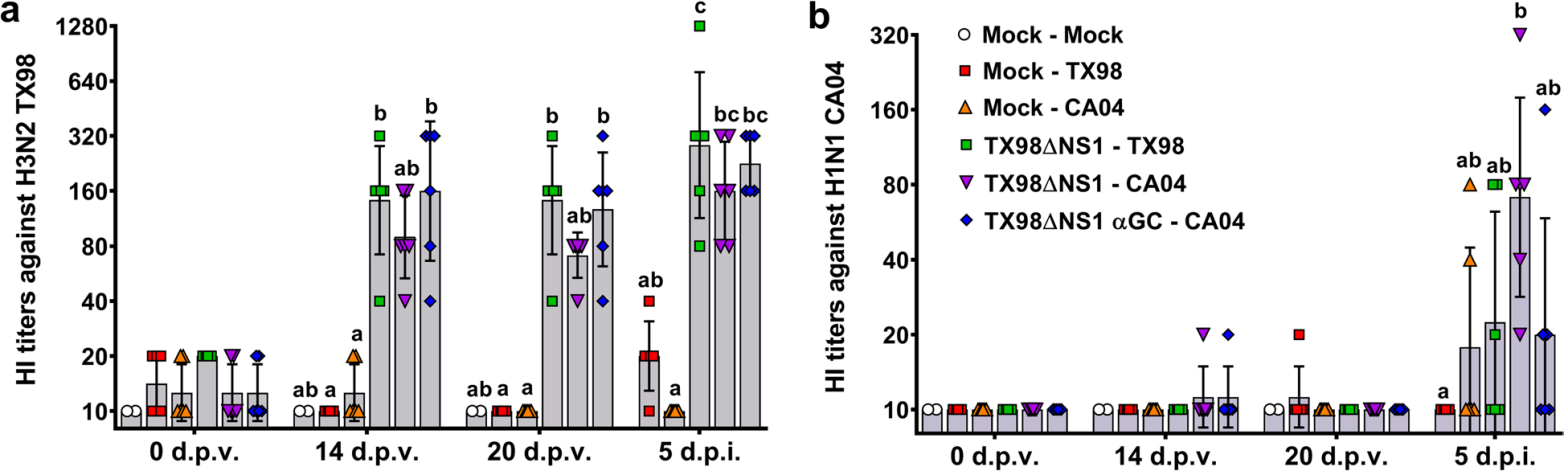
Virus-specific antibody titers. **a**, **b** Geometric mean of hemagglutination inhibition titers against TX98 (**a**) and CA04 (**b**) antigens in sera collected at 0, 14, and 20 d.p.v., and 5 d.p.i.. Differences between treatments were analyzed using a Dunn’s multiple comparison test. A statistically significant difference between two groups is indicated by different letters. Data are represented as geometric mean ± geometric standard deviation. Symbols represent individual pigs

Vaccination induced moderate TX98-specific HI titers by 14 d.p.v. that was maintained until the end of the challenge period (5 d.p.i.) (Fig. 3a). Vaccination did not induce CA04-specific HI titers during the 21-day vaccination period. After infection, the highest CA04-specific HI titers were in CA04-infected pigs vaccinated with the H3N2 LAIV but without α-GalCer treatment (Fig. 3b). Pigs vaccinated with α-GalCer tended to have lower CA04-specific HI titers than pigs that received the LAIV vaccine alone (Fig. 3b). These results indicate that the TX98ΔNS1 LAIV vaccine had a modest capacity to induce cross-reactive CA04-specific antibodies, and that the concentration of these antibodies was numerically reduced by combining α-GalCer with the LAIV vaccine.

#### Cellular responses

**Fig 4:**
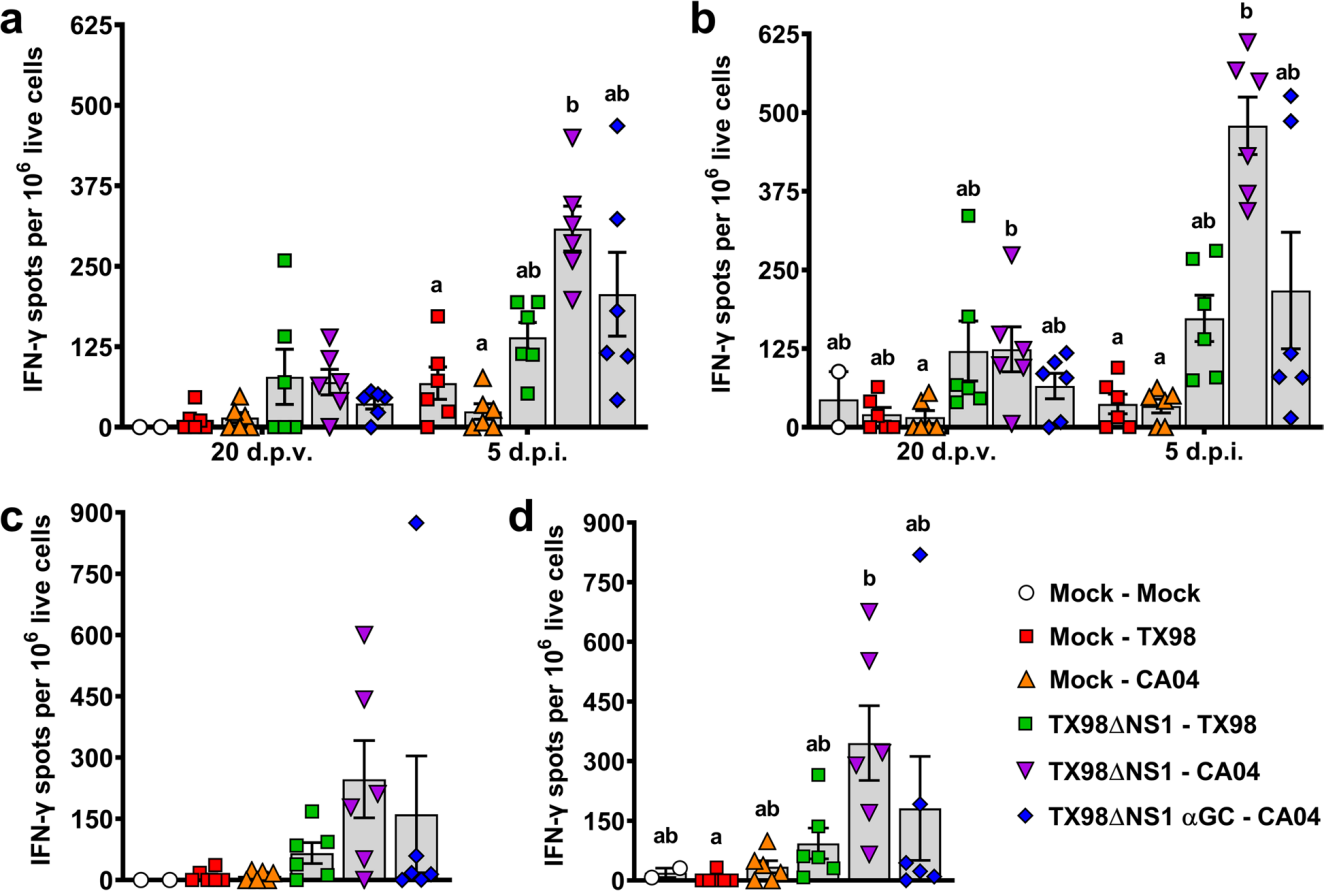
Cellular responses to TX98 and CA04 measured by IFN-γ ELISPOT assays. IFN-γ production by PBMC collected at 20 d.p.v. and 5 d.p.i. after incubation with UV-inactivated TX98 (**a**) or CA04 (**b**) virus particles. IFN-γ production by lung leukocytes isolated at 5 d.p.i. after incubation with UV-inactivated TX98 (**c**) or CA04 (**d**) virus particles. Results represent mean IFN-γ spots per 1x10^6^ live cells after subtracting spots counted in unstimulated wells. Differences between treatments were analyzed by Dunn’s multiple comparisons test. A statistically significant difference between two groups is indicated by different letters. Data are represented as mean ± SEM. Symbols represent individual pigs

Interferon-γ enzyme-linked immune absorbent spot (ELISPOT) assays were performed to determine the effect of vaccination and α-GalCer on homologous and heterosubtypic cellular immune responses. Unvaccinated pigs did not develop measurable TX98- or CA04-specific peripheral blood mononuclear cells (PBMC) until 5 d.p.i.. In contrast, LAIV-vaccinated pigs started presenting TX98- and CA04-reactive PBMC by 20 d.p.v. (Fig. 4a and b). In pigs that received the vaccine without α-GalCer, infection with TX98 induced a modest increase in CA04-reactive cells, and had no effect on the frequency of TX98-reactive cells, compared to unvaccinated pigs. In contrast, we found a high frequency of both TX98- and CA04-specific immune cells in pigs which received the LAIV vaccine without α-GalCer that were infected with CA04. Interestingly, pigs vaccinated with α-GalCer tended to have fewer TX98- and CA04-reactive cells than pigs administered the vaccine alone. Similar results were obtained in the lung where lower concentrations of TX98- and CA04-reactive cells were detected in pigs vaccinated with α-GalCer compared to pigs that received the vaccine alone (Fig. 4c and d). Collectively, these data showed that vaccination with the TX98ΔNS1 LAIV vaccine induced heterosubtypic cellular responses against CA04, and that α-GalCer seemed to diminish these responses.

#### Replication of vaccine and challenge viruses

**Fig 5:**
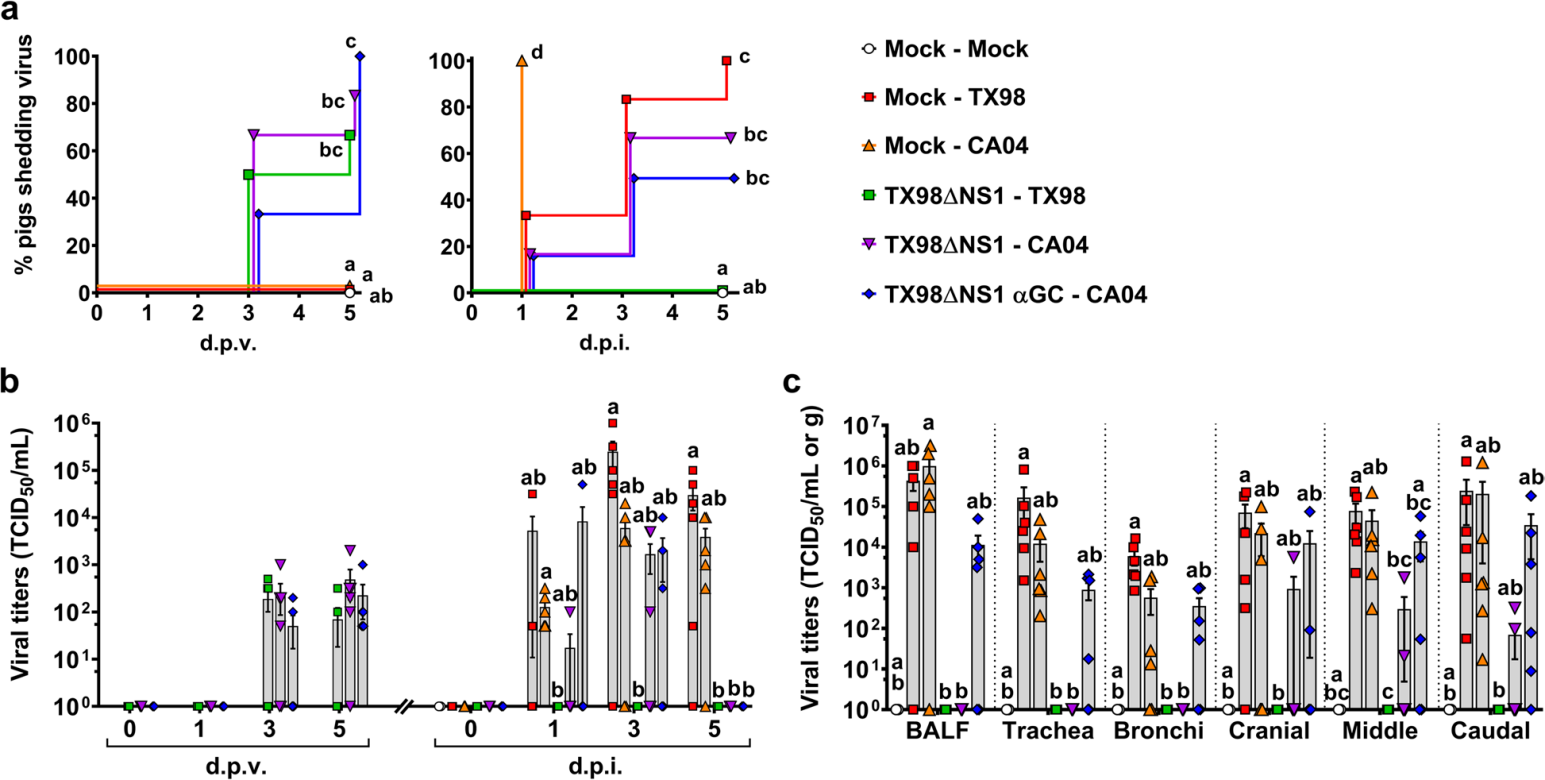
Viral titers in nasal swabs and respiratory tissues. **a** Percentage of pigs positive for virus shedding in nasal swabs collected at 0, 1, 3, and 5 d.p.v. and 0, 1, 3, and 5 d.p.i.. **b** Virus titers in nasal swabs after vaccination and infection. **c** Virus titers in BALF and homogenized respiratory tissues at 5 d.p.i.. Data are represented as TCID_50_/mL for nasal swabs and BALF and TCID_50_/g for respiratory tissues. Differences between treatments were analyzed by Mantel-Cox log-rank test (**a**), or Dunn’s multiple comparisons test (**b**, **c**). A statistically significant difference between two groups is indicated by different letters. Data are represented as mean ± SEM. Symbols represent treatment groups (**a**) or individual pigs (**b**, **c**)

All three vaccinated groups started shedding the LAIV by 3 d.p.i.. Pigs treated with α-GalCer shed similar levels of TX98ΔNS1 to the other vaccinated groups (Fig. 5a and b). After challenge, unvaccinated pigs shed high levels of TX98 and CA04. However, while all unvaccinated CA04-infected pigs shed virus by 1 d.p.i., it took longer for unvaccinated TX98-infected pigs to shed virus, with 5 out of 6 pigs positive by 3 d.p.i. and one pig shedding only at 5 d.p.i. (Fig. 5a). No virus was detected in the nasal swabs of vaccinated pigs challenged with TX98. Vaccinated pigs challenged with CA04 shed similar levels of virus to unvaccinated pigs at 1 and 3 d.p.i.. However, these pigs stopped shedding virus by 5 d.p.i., regardless of whether they received α-GalCer or not (Fig. 5a and b). Unvaccinated pigs had high titers of TX98 and CA04 in BALF, trachea, bronchi, and lung tissues at 5 d.p.i. (Fig. 5c). No virus was detected in the BALF or respiratory tissues of vaccinated pigs challenged with TX98. Similarly, no virus was detected in the BALF, trachea, or bronchi of CA04-challenged pigs that had been vaccinated without α-GalCer. Moreover, only three of these pigs had virus positive lung samples. Conversely, virus was present in the respiratory tissues and BALF of all but one of the α-GalCer treated pigs, albeit at lower titers than the sham-vaccinated pigs. In summary, these results show that vaccination with TX98ΔNS1 inhibited replication of the heterosubtypic CA04 virus and that this effect was reduced by combining the LAIV vaccine with α-GalCer.

#### Lung pathology

**Fig 6:**
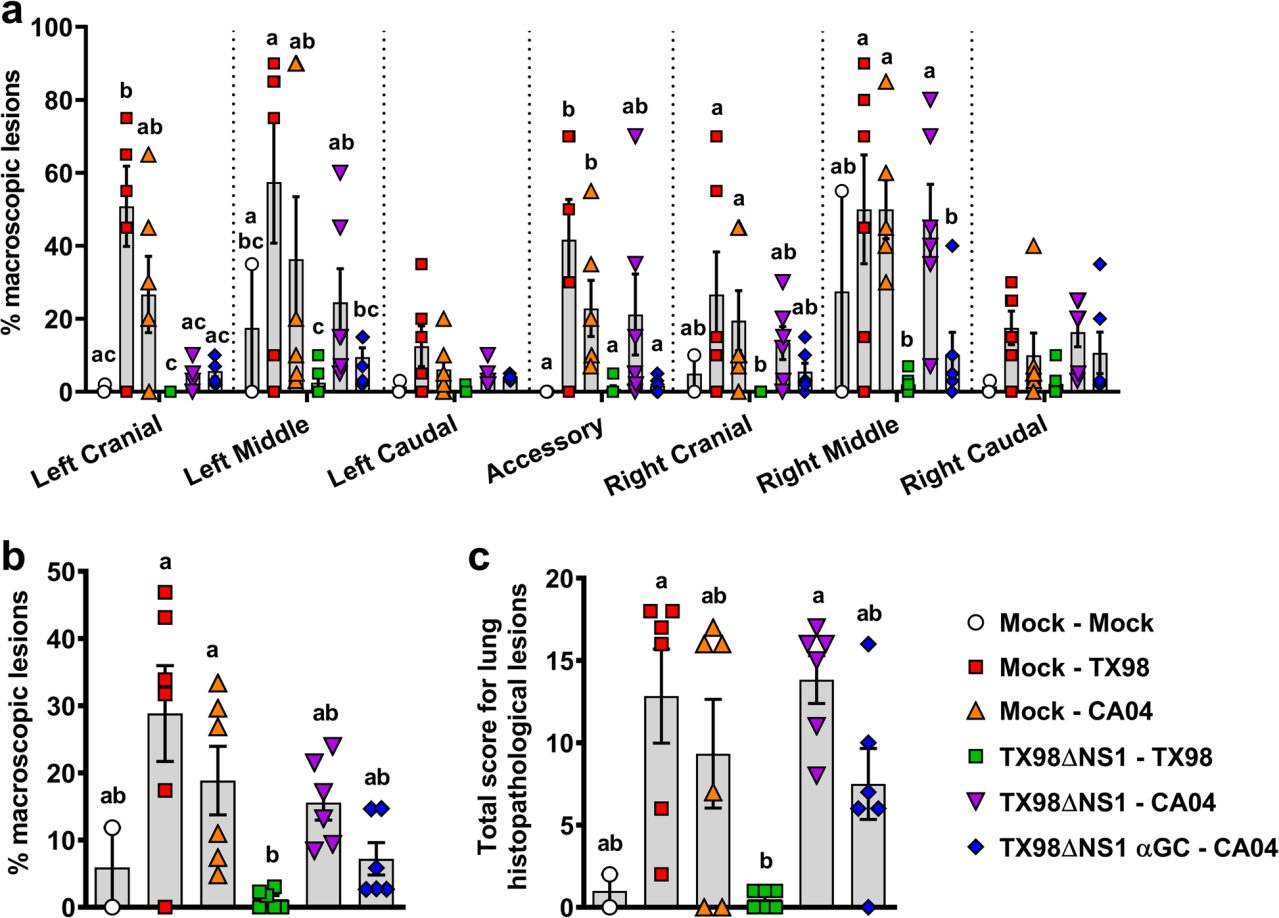
Macroscopic lung lesion scores and histopathology at 5 d.p.i.. **a**, **b** Macroscopic lesions assessed in (**a**) individual lung lobes and (**b**) total lungs according to the relative volume of each lobe. **c** Histopathology scores assessed by H&E staining according to the materials and methods. Differences between treatments were analyzed by Tukey’s (**a**) or Dunn’s (**b**, **c**) multiple comparisons test. A statistically significant difference between two groups is indicated by different letters. Data are represented as mean ± SEM. Symbols represent individual pigs

Macroscopic lung pathology was evaluated at necropsy according to the percentage of individual lung lobes or total lung surface area affected by atelectasis and pneumonia (Fig. 6a and b). Unvaccinated pigs challenged with TX98 had the highest percentage of surface area affected by disease, followed by unvaccinated animals challenged with CA04. Vaccinated pigs challenged with TX98 had very few lung lesions. In contrast, pigs vaccinated without α-GalCer and challenged with CA04 had high levels of atelectasis and pneumonia that were comparable in most lung lobes to the unvaccinated pigs (Fig. 6a). An exception was the left cranial lung lobe in which the vaccinated pigs without α-GalCer had fewer macroscopic lesions compared to the unvaccinated pigs. Combining the vaccine with α-GalCer reduced CA04-induced macroscopic lung lesions to approximately half the level of pigs that received the vaccine alone (Fig. 6b). Similar results were obtained from a histopathological assessment of microscopic lung lesions (Fig. 6c). Together, these data indicate that the LAIV vaccine alone did not significantly impact lung pathology induced by CA04 infection. However, adjuvanting the vaccine with α-GalCer led to a numerical reduction in lung inflammation scores, which may be related to the lower concentrations of virus-reactive cells found in these pigs.

## Discussion

Here, we assessed the feasibility of using α-GalCer to increase the heterologous and heterosubtypic cross-protection afforded by a LAIV vaccine formerly used by the US swine industry (Ingelvac Provenza™; Boehringer Ingelheim Animal Health USA, Inc., Duluth, GA) (Genzow et al. 2018; Sharma et al. 2020). Our premise was based on a previous report which found that mice intranasally administered a combination of α-C-GalCer and a similar LAIV vaccine had a substantially improved rate of survival compared to mice that received the LAIV alone, after a lethal infection with a homologous virus (Kopecky-Bromberg et al. 2009). Like our swine LAIV vaccine (Solórzano et al. 2005), this LAIV was produced using an eight-plasmid reverse genetic system including a plasmid encoding a truncated NS1 protein (Kopecky-Bromberg et al. 2009). Viruses with this truncation are highly attenuated because NS1 is required to inhibit host interferon responses (García-Sastre et al. 1998; Fernandez-Sesma et al. 2006; Richt and García-Sastre 2009). The TX98ΔNS1 LAIV vaccine provides pigs complete immunity against the homologous wild-type virus and partial protection against heterologous and heterosubtypic IAV strains (Solórzano et al. 2005; Richt et al. 2006; Vincent et al. 2007).

In our first experiment to identify dosages of α-GalCer that avoid compromising LAIV growth, we found indications that α-GalCer inhibited LAIV replication and that the 100 μg/kg dose tended to reduce protection from lung disease. α-GalCer-mediated reductions in LAIV levels did not suppress the capacity of the vaccine to inhibit replication of the heterologous challenge virus. This may be because the TX98ΔNS1 LAIV vaccine is highly effective against CO99 (Vincent et al. 2007) and probably remains effective at quite low doses. Decreased levels of LAIV in α-GalCer treated pigs is consistent with reports that α-GalCer treatment significantly reduces virus levels in IAV-infected mice (Ho et al. 2008; De Santo et al. 2008). This has been associated with several NKT cell-mediated innate immune responses, including the induction of type I (IFN-α, IFN-β) and II (IFN-γ) interferons, recruitment of NK cells to the infection site, and reduction of the suppressive activity of myeloid cells (Ishikawa et al. 2010; Ho et al. 2008; De Santo et al. 2008). Our results agree with the findings of Kopecky-Bromberg et al. (2009), who observed that high doses of α-C-GalCer eliminated the protection afforded by a LAIV vaccine. It also agrees with our previous study which found that 100 μg/kg of α-GalCer intranasally administered to IAV-infected pigs reduced virus titers in nasal swabs and lung tissue (Artiaga et al. 2016b).

Our second experiment investigated whether adjuvanting the LAIV TX98ΔNS1 with 50 μg/kg of α-GalCer would increase protection against a heterosubtypic virus challenge. We found that, even though the amount of LAIV shedding in nasal swabs was not reduced, this dose of α-GalCer diminished the LAIV vaccine’s ability to induce cross-protective immune responses and to inhibit virus replication. An interesting observation was that pigs vaccinated with α-GalCer had lower levels of lung pathology compared to animals that received the LAIV vaccine alone. This may partly be due to lower concentrations of virus-specific T cells in the lungs of α-GalCer vaccinated pigs, since the accumulation of inflammatory cells in airway tissue is an important contributer to pulmonary inflammation (Humphreys et al. 2003; Paget et al. 2011; Duan and Thomas 2016). The LAIV vaccine alone had no effect on CA04-induced lung pathology, which matches a previous report that challenged TX98ΔNS1-vaccinated pigs with a different heterosubtypic virus strain (Vincent et al. 2007).

On a body weight basis, the 50 μg/kg dose of α-C-GalCer is comparable to the 1 μg/mice dose that Kopecky-Bromberg et al. (2009) used to increase the survival of LAIV vaccinated mice (Kopecky-Bromberg et al. 2009). Several factors may have contributed to why we did not observe a similar pattern of protection. Firstly, the mouse study used a derivative of α-GalCer, i.e. α-C-GalCer, that induces enhanced and prolonged production of IFN-γ compared to α-GalCer (Schmieg et al. 2003; Fujii et al. 2006). Secondly, our study tested the effectiveness of α-GalCer for stimulating heterosubtypic immune responses, which are more difficult to induce compared to the homologous vaccine-challenge regimen used by Kopecky-Bromberg et al. (2009). Thirdly, NKT cell concentrations are much lower in pigs than in most inbred mouse strains and the tissue distribution and subsets of mouse NKT cells differ substantially from pigs (Artiaga et al. 2014; Yang et al. 2017; Lee et al. 2015). Fourthly, mice are not natural hosts of IAVs and usually develop more severe clinical disease than pigs, but without IAV-specific clinical signs (Francis 1934; Francis and Magill 1935). This is largely due to differences in mouse and porcine antiviral immune defenses that include several non-orthologous antiviral genes with relatively low sequence similarity (Pillai et al. 2016; Starbæk et al. 2018). Lastly, the intranasal route of α-GalCer delivery is probably more efficient at stimulating mucosal NKT cells in the respiratory tract of mice compared to pigs as the relative distance between the nasal passages and the lungs is considerably shorter in mice than in pigs. Since pigs are anatomically and immunologically more similar to humans than to mice and are also natural hosts of IAVs, it is quite likely that swine more accurately reflect how humans would respond to α-GalCer as a LAIV vaccine adjuvant.

## Conclusions

Together, our results found that adjuvanting LAIV vaccines with α-GalCer weakened rather than enhanced immunity against a hetersubtypic virus challenge. This was likely due to NKT cell-mediated innate responses that inhibited growth of the LAIV vaccine. It is possible that using lower levels of α-GalCer would overcome this obstacle. However, since there is substantial heterogeneity in NKT cell frequencies and effector functions among pigs and humans, the danger exists that even very low doses of α-GalCer will inhibit LAIV growth in some individuals.

## Methods

### Pigs

Four-week-old pigs of mixed breed and sex were acquired from Midwest Research Swine Inc. (Glencoe, MN) and transported to Kansas State University’s Large Animal Research Facility (Manhattan, KS). The animals were allowed to acclimatize to the research facility for 3 days before being enrolled in the experiments. Hemagglutination inhibition (HI) assays and RT-qPCR were respectively used to confirm that pigs were seronegative for H1/H3 antibodies and virus shedding as previously described (Kitikoon et al. 2014; Sponseller et al. 2010).

### Virus and vaccine preparation

The LAIV vaccine was initially generated by reverse genetics from H3N2 A/Swine/Texas/4199-2/1998 (TX98) influenza virus as described previously (Solórzano et al. 2005). Briefly, the LAIV encodes a truncated NS1 protein with four stop codons introduced after 126 reading codons, resulting in a 3’ truncation of the wild-type NS1 protein from 219 to 126 amino acids. The remaining genetic material from wild-type TX98 was used to encode PB2, PB1, PA, HA, NP, NA, M1, M2 and NS2. Plasmids encoding each gene segment were used to transfect HEK 293T human embryonic kidney cells expressing a temperature-sensitive mutant of SV40 large T antigen using the TranslT®-LT1 transfection reagent (Mirus Bio LLC, Madison, WI). The HEK 293T cells were subsequently co-cultured with Madin-Darby Canine Kidney (MDCK) cells, after which virus particles recovered from the culture supernatant were further propagated through MDCK cells. For the current studies, the LAIV vaccine and challenge viruses were propagated through MDCK cells from in-house stocks. The challenge viruses included the wild-type TX98 containing an intact NS1 gene, the H3N2 A/Swine/Colorado/23619/1999 (CO99), and the H1N1 pandemic A/California/04/2009 (CA04) viruses. The identity of the virus subtypes was confirmed by Sanger sequencing.

### Virus titration

Nasal swabs and BALF were collected in DMEM (Corning, Corning, NY) supplemented with antibiotic-antimycotic (Gibco Life Technologies, Carlsbad, CA), filtered using a 0.45 μm syringe-filter, and stored at -80°C. Trachea, bronchi, and lung were mechanically dissociated in DMEM supplemented with 0.3% Bovine Serum Albumin (BSA, Sigma-Aldrich, St. Louis, MO), 1⨯ MEM Vitamin (Gibco), and 1⨯ antibiotic-antimycotic (Gibco) using a TissueLyser II (Qiagen, Germantown, MD) and stainless-steel beads. The resulting tissue homogenates were filtered through 0.45 μm cell strainers and stored at -80°C until further processing.

Viral titers were determined by median (50%) of tissue culture infectious dose (TCID_50_) and expressed as log transformed value of TCID_50_/mL or TCID_50_/g, as appropriate. Briefly, the TCID_50_ values were determined by infecting MDCK cells in 96-well microtiter plates with serial dilutions of virus. Samples were incubated at 37°C with 5% CO_2_ for 48 hours in infection media (DMEM + 0.3% BSA + MEM Vitamin + antibiotic-antimycotic) supplemented with 1 μg/mL of L-(tosylamido-2-phenyl) ethyl chloromethyl ketone (TPCK)-treated trypsin (Worthington Biochemical Corporation, Lakewood, NJ). For tissue homogenate samples, serial dilutions media was changed after 3 hours to fresh infection media with TPCK-treated trypsin. Plates containing nasal swab and BALF samples were fixed with methanol for 10 minutes at -20°C and stained using monoclonal antibodies against influenza A nucleoprotein (NP) (HB65 hybridoma ATCC, Manassas, Virginia) and subsequently incubated with rabbit anti-mouse immunoglobulin secondary antibody conjugated to horseradish peroxidase (HRP) (Dako, Glostrup, Denmark), and 3-amino-9-ethylcarbazole substrate (AEC) (Electron Microscopy Sciences, Hatfield, PA). Tissue homogenate samples were processed in the same way except that an Alexa Fluor 488-conjugated polyclonal goat anti-mouse IgG (Invitrogen, Carlsbad, CA) was used as the secondary antibody, so that the samples could be read by indirect immunofluorescence. TCID_50_ values were calculated by the method of Reed and Muench (Reed and Muench 1938).

### Experimental design

In Experiment 1, 15 pigs were assigned to five treatment groups of three pigs each (Table 1 and Additional Fig. 1a). On day 0, pigs in groups 2-5 were intranasally administered 2 mL DMEM (1 mL per nostril) containing 10^6^ TCID_50_ TX98ΔNS1, combined with either 0 (vehicle only), 10, 50, or 100 μg/kg of α-GalCer (Diagnocine LLC Hackensack, NJ). Stock solutions of α-GalCer (2 mg/mL) were dissolved in DMSO as previously described (Artiaga et al. 2014). Pigs in group 1 were sham-vaccinated with 50 μL/kg DMSO dissolved in 2 mL of DMEM, which was the volume of DMSO used to dissolve the 100 μg/kg dose of α-GalCer. Twenty-eight days after inoculation, pigs were sedated with an intramuscular injection of tiletamine-zolazepam (Telazol®; 4.4 mg/kg of body weight) and xylazine (2.2 mg/kg) and i.t. infected with 10^6^ TCID_50_ CO99 in 2 mL of DMEM. Body temperature and clinical signs were assessed at -2, 0, 1, 3, 5, 7, 14, and 20 d.p.v. and daily throughout the challenge period. Peripheral blood was collected at -2, 20, and 33 d.p.v. to analyze immune cell populations by flow cytometry. Serum was collected on days -2, 7, 14, 20, 28, and 33 d.p.v. to assess virus-specific antibodies by HI assay. Nasal swabs were collected at -2, 1, 3, and 5 d.p.v. and daily during the challenge period to assess virus shedding in nasal secretions. At 5 d.p.i. (33 d.p.v.), pigs were sedated with tiletamine-zolazepam and xylazine and euthanized with a lethal dose of Pentobarbital Sodium IV injections (150 mg/kg of body weight). Bronchioalveolar lavage fluid was collected by lavaging the lung with 50 mL of DMEM. Lung tissue and TBLN were collected into DMEM for analysis of immune cells by flow cytometry. The right middle lung lobe was collected into formalin for histopathological analysis. One pig in group 2 died from anesthesia complications at the time of infection and was removed from the analysis.

In Experiment 2, 32 pigs from 4 litters were assigned to 6 treatment groups so that each group contained a similar number of pigs from each litter (Table 2; Additional Fig. 1b). On day 0, the pigs were intranasally vaccinated using the same protocol employed in Experiment 1. Groups 1, 2, and 3 were sham-vaccinated. Groups 4 and 5 received 10^6^ TCID_50_ TX98ΔNS1. Group 6 received the same dose of LAIV vaccine combined with 50 μg/kg of α-GalCer. Three weeks after vaccination (21 d.p.v.), groups 2 and 4 were i.t. infected with wild-type 10^6^ TCID_50_ TX98 in 2 mL of DMEM, while groups 3, 5, and 6 were infected with the same dose of CA04. On the same day, group 1 was euthanized and necropsied as described in Experiment 1. All the remaining groups were necropsied at 5 d.p.i. (26 d.p.v.). Body temperature, clinical signs, viral titers, histopathology, immunological analyses, and serological analyses were performed identically to Experiment 1 with the exception that trachea, bronchi, and lung lobes were also collected for viral titers.

### Tissue processing for single cell isolation

Single cells from peripheral blood, BALF, lung tissue, and TBLN were isolated and prepared for flow cytometry and ELISPOT assays as previously described (Artiaga et al. 2014; Artiaga et al. 2016a). Briefly, blood samples were collected by venipuncture from the jugular vein into vacutainer tubes coated with EDTA or heparin (BD Biosciences, San Jose, CA), and tissue samples were collected into DMEM. Peripheral blood was treated with an ammonium chloride-based lysis buffer to remove red blood cells (RBC). Peripheral blood mononuclear cells were isolated by density gradient centrifugation using Ficoll-Paque™ PREMIUM (GE Healthcare BioSciences Corp., Uppsala, Sweden) as previously described (Artiaga et al. 2014). Cells were then resuspended in freezing media [45% RPMI 1640 (ATCC), 45% fetal bovine serum (FBS; Atlanta Biologicals, Flowery Branch, GA) and 10% DMSO (Sigma-Aldrich) and slowly frozen in a freezing container with isopropanol at -80°C for 24 hours, before being transferred to liquid nitrogen. The BALF samples were centrifuged and the cell pellets and supernatants collected to respectively analyze immune cells and viral titers. Approximately 2 grams of lung tissue sampled from cranial, middle, and caudal lobes were digested with 5 μg/mL of Liberase TL (Roche Diagnostics Deutschland GmbH, Mannheim, Germany) in DMEM at 37°C for 45 minutes, passed through a 100 μm cell strainer (Fisher Scientific), and treated with the above-mentioned RBC lysis buffer. TBLN was homogenized into single cell suspensions using disposable tissue grinders (Fisher Scientific, Pittsburgh, PA), filtered using a 100 μm cell strainer, and treated with RBC lysis buffer. Single cells were resuspended in PBS and stained with 0.4% trypan blue to count total cells and viability using a Countess™ II Automated Cell Counter (Life Technologies).

### Flow cytometry and antibodies

Cell suspensions were incubated with a viability dye (LIVE/DEAD™ Fixable Near-IR Dead Cell Stain Kit, Invitrogen) for exclusion of dead cells, Fc blocked using a 1 mg/mL solution of rat IgG (Sigma-Aldrich), and stained with the indicated monoclonal antibodies (Abs) at 4°C. T cell and NK cell subsets were distinguished using Abs specific for CD3ε (BB23-8E6-8C8; BD Biosciences), CD4 (74-12-4; Southern Biotech, Birmingham, AL), CD8α (76-2-11; Southern Biotech), CD8β (PPT23; Bio-Rad, Hercules, CA), TCRδ (PGBL22A; WSU Monoclonal Antibody Center, Pullman WA), CD16 (G7; BD Biosciences), and CD11b (M1/70; BioLegend). NKT cells were identified using a PBS57-loaded mouse CD1d tetramer and an unloaded CD1d control tetramer from the National Institutes of Health Tetramer Core Facility. Monocytes, macrophages, and granulocytes were characterized using Abs specific for CD14 (MIL2; Bio-Rad), CD16, CD163 (2A10/11; Bio-Rad), CD172a (74-22-15A; BD Biosciences), CD11b, and MHC class II (H42A; WSU Monoclonal Antibody Center) (Additional Table 1 and Additional Figure 2). Stained cells were washed once with PBS, fixed using the BD Cytofix/Cytoperm kit (BD Biosciences), and washed once more with PBS before being acquired using a BD LSRFortessa™ X-20 flow cytometer with FACSDiva software (version 8.0.1, BD Biosciences). Fluorescence-minus-one controls were used to determine positive and negative populations. All data were analyzed using FlowJo software (version 10.7.0, Treestar, Palo Alto, CA).

### ELISPOT assay

Frozen PBMC were thawed in a water bath at 37°C, washed twice with thawing media (RPMI 1640 and 20% FBS), resuspended in culture media [RPMI 1640, 10% FBS, 1% antibiotic-antimycotic, and 55 μM 2-mercaptoethanol (Gibco)], and rested for 2 hours. Single cells isolated after lung digestion were not cryopreserved but used immediately. PBMC or lung cells were plated at 0.5 or 1 million live cells per well in 96 well MultiScreen HTS plates (Millipore, Billerica, MA) pre-coated with anti-IFN-γ (P2G10, BD Biosciences). The cells were then incubated at 37°C for 48 hours with 5 x 10^5^ TCID_50_ of UV-inactivated TX98 or CA04 virus particles or virus-free UV-treated MDCK supernatant. Afterwards, the plates were developed using a biotin-conjugated anti-IFN-γ mAb (P2C11, BD Biosciences), streptavidin-HRP (BD Biosciences), and AEC substrate (BD Biosciences), according to manufacturer instructions. The number of spots in each well was read using an ImmunoSpot S6 Micro Analyzer ELISPOT reader with ImmunoCapture 6.4 software (Cellular Technology Ltd., Shaker Heights, OH). The data are presented as the number of spots per 10^6^ PBMC or lung cells after subtracting the average number of spots in wells cultured with virus free MDCK supernatant.

### HI assay

Hemagglutination inhibition assays were performed on serum samples treated overnight at 37°C with receptor destroying enzyme II (Denka Seiken, Tokyo, Japan), heat inactivated at 56°C for 60 min, and incubated with 0.5% washed chicken RBC (Colorado Serum Company, Denver, CO) at 4°C for 60 min to remove non-specific agglutinants. This treatment results in samples being diluted 1:10 from the original sample, after which they were serially diluted at 1:2 with PBS. HI assays were performed using 4 HA units of TX98, CO99, or CA04 viruses as antigens and 0.5% washed chicken RBC as previously described (Kitikoon et al. 2014). The highest sample dilution that inhibited virus-induced RBC hemagglutination is presented.

### Pathology and histopathology

At necropsy, the lungs were removed from the thoracic cavity and assessed for the percentage of the surface area affected by red and depressed areas (atelectasis), which is characteristic of IAV-induced pneumonia. The percentage of each lung lobe affected by pneumonia was visually estimated and a total score was then calculated for each pig based on the relative proportion of each lung lobe to the total lung: The right and left cranial and middle lobes were assigned as 10% each, the accessory lobe was assigned 5%, and the right and left caudal lobes were assigned 27.5% each for a total of 100% (Halbur et al. 1995). The right middle lung lobe, which tended to have the highest lesion scores was collected and fixed in 10% neutral phosphate-buffered formalin, embedded in paraffin, and stained with hematoxylin and eosin. Two sections of lung were blindly scored for histopathological lesions. A previously described rubric was used to score each lung section from 0 to 3 for 6 separate criteria typically associated with IAV infections in pigs: (i) epithelial necrosis, attenuation or disruption; (ii) airway exudate-necrosis/inflammation; (iii) percentage of airways with inflammation; (iv) peribronchiolar and perivascular lymphocytic inflammation; (v) alveolar exudate; (vi) alveolar septal inflammation (Khurana et al. 2013). The total sum of the scores was calculated for each pig.

### Statistical analysis

Data were graphed and analyzed using GraphPad Prism version 9.3.1 (GraphPad Software, San Diego, CA). The normality of the data was evaluated by the Shapiro-Wilk test. Data for body temperature changes, NKT cell frequencies, and macroscopic lung lesion scores per lobe were normally distributed and evaluated using a one-way or two-way analysis of variance (ANOVA). Means were separated using Turkey’s multiple comparison test when a main effect or interaction term was determined to be significant (*P* < 0.05). Data for HI titers, IFN-γ ELISPOT assays, viral titers, macroscopic lung lesion scores for total lung, and histopathological lesion scores were not normally distributed and therefore analyzed using a nonparametric Kruskal-Wallis test and a Dunn’s multiple comparisons test. Survival curves were analyzed by Mantel-Cox log-rank test.

## Supplementary Information

**Additional file 1: Additional Figure 1.** Timelines for the experiments. **Additional Table 1.** Reagents used for flow cytometry. **Additional Figure 2.** Gating strategy to identify immune cell populations in peripheral blood, BALF, and tissues.

## Data Availability

All data generated during this study are included in this published article and additional material file. Additional Fig. 1: Timelines for the experiments; Additional Fig. 2: Gating strategy to identify immune cell populations in peripheral blood, BALF, and tissues; Additional Table 1: Reagents used for flow cytometry.

## Abbreviations

Abs: Antibodies
AEC: 3-amino-9-ethylcarbazole
AF: Alexa Fluor
ANOVA: Analysis of variance
BALF: Bronchioalveolar lavage fluid
BSA: Bovine serum albumin
BV: Brilliant Violet
CA04: H1N1 A/California/04/2009 influenza A virus
CD: Cluster of differentiation
CO99: H3N2 A/Swine/Colorado/23619/1999 influenza A virus
Cy: Cyanine
d.p.i.: Days post infection
d.p.v.: Days post vaccination
DC: Dendritic cell
DMEM: Dulbecco's modified Eagle's medium
DMSO: Dimethyl sulfoxide
EDTA: Ethylenediaminetetraacetic acid
ELISPOT: Enzyme-linked immune absorbent spot
FBS: Fetal bovine serum
HA: Hemagglutinin
HEK 293T: Transfectable derivative of human embryonic kidney 293 cells
HI: Hemagglutination inhibition
HRP: Horseradish peroxidase
i.n.: Intranasally
i.t.: Intratracheally
IACUC: Institutional Animal Care and Use Committee
IAV: Influenza A virus
IBC: Institutional Biosafety Committee
IFN: Interferon (IFN-α, IFN-β, IFN-γ)
Ig: Immunoglobulin
LAIV: Live-attenuated influenza virus
MDCK: Madin-Darby canine kidney
MHC: Major histocompatibility complex
N/A: Not applicable
NK: Natural killer
NKT: Natural killer T
NP: Nucleoprotein
NS: Not significant
NS1: Non-structural protein 1
PBMC: Peripheral blood mononuclear cell
PBS: Phosphate buffered saline
PBS57: Analogue of α-GalCer developed by Dr. Paul Savage and colleagues
PE: R-phycoerythrin
PerCP: Peridinin chlorophyll protein
RBC: Red blood cell
RDE II: Receptor destroying enzyme II
RPMI 1640: Roswell Park Memorial Institute culture media 1640
RT-qPCR: Quantitative reverse transcription polymerase chain reaction
SEM: Standard error of the mean
TBLN: Tracheobronchial lymph node
TCID50: Median (50%) of tissue culture infectious dose
TCR: T cell receptor
TPCK: L-(tosylamido-2-phenyl) ethyl chloromethyl ketone
TX98ΔNS1: H3N2 A/Swine/Texas/4199-2/1998 influenza A virus with a truncated NS1 protein
TX98: H3N2 A/Swine/Texas/4199-2/1998 influenza A virus
UV: Ultra-violet
α-C-GalCer: Alpha-C-galactosylceramide
α-GalCer: Alpha-galactosylceramide
