# Supplementary material for: Evaluating α-galactosylceramide as an adjuvant for live attenuated influenza vaccines in pigs
Source: Anim Dis. 2022 Aug 1;2(1):19. doi: 10.1186/s44149-022-00051-x (PMC9339466; doi:10.1186/s44149-022-00051-x)
Supplement: Supplementary file 1 — Additional file 1: Additional Figure 1. Timelines for the experiments. Additional Table 1. Reagents used for flow cytometry. Additional Figure 2. Gating strategy to identify immune cell populations in peripheral blood, BALF, and tissues. [file 44149_2022_51_MOESM1_ESM.docx]

**Title**

Evaluating α-galactosylceramide as an adjuvant for live attenuated influenza vaccines in pigs

**Authors**

Bianca L. Artiaga^1^, Igor Morozov^1^, Russell Ransburgh^1^, Taeyong Kwon^1^, Velmurugan Balaraman^1^, Sabarish V. Indran^1^, Darling Melany De Carvalho Madrid^2^, Weihong Gu^3^, Jamie Henningson^1^, Wenjun Ma^1^, Jürgen A. Richt^1^, John P. Driver^2*^

**Affiliations**

^1^ Department of Diagnostic Medicine & Pathobiology, College of Veterinary Medicine, Kansas State University, Manhattan, KS 66506, USA;

^2^ Division of Animal Sciences, University of Missouri, Columbia, MO 65211, USA;

^3^ Department of Animal Sciences, University of Florida, Gainesville, FL 32611, USA

* Corresponding author: J.P.D. driverjp@missouri.edu

**Additional Material**

**
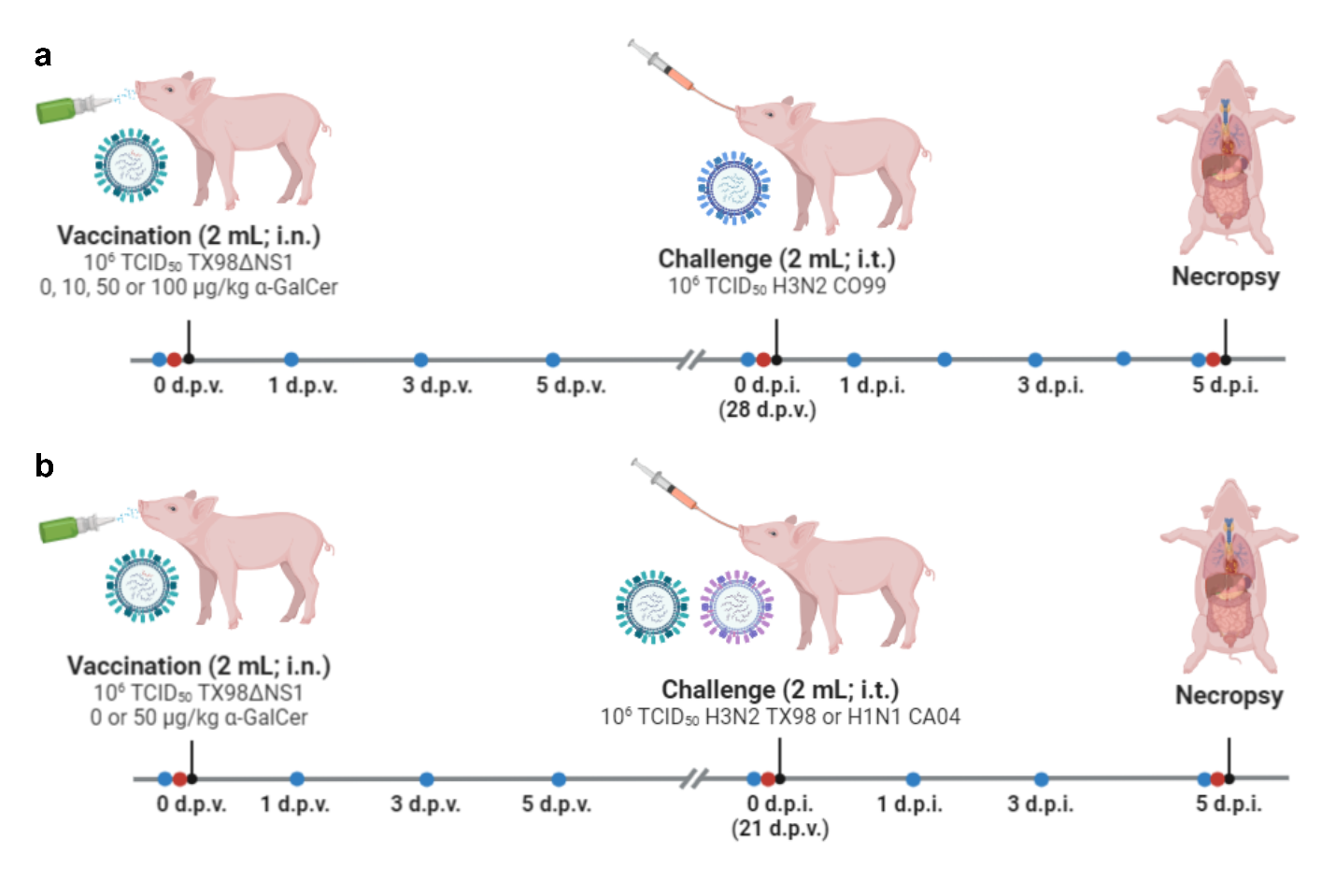
Additional Figure 1.**  Experimental setup. (a) In Experiment 1, pigs were vaccinated with TX98ΔNS1 in combination with different doses of α-GalCer and challenged 28 days later with CO99. Necropsies were performed at 5 d.p.i.. (b) In Experiment 2, pigs were vaccinated with or without 50 ug/kg α-GalCer and challenged 21 days later with TX98 or CA04. Necropsies were performed at 5 d.p.i.. Blood and nasal swab collections are respectively represented by red and blue circles. TX98ΔNS1: H3N2 A/Swine/Texas/4199-2/1998 encoding a truncated NS1 protein; H3N2 CO99: H3N2 A/Swine/Colorado/23619/1999; H3N2 TX98: H3N2 A/Swine/Texas/4199-2/1998; H1N1 CA04: H1N1 A/California/04/2009; i.n.: intranasal; i.t.: intratracheal. Figures created with BioRender.com.

**Additional Table 1.** Reagents used for flow cytometry analysis of surface markers.

| **Marker** | **Clone** | **Isotype** | **Source** | **Fluorochrome** | **Conjugation in house** |
| --- | --- | --- | --- | --- | --- |
| CD3ε | BB23-8E6-8C8 | Mouse IgG2aκ | BD Biosciences | PE-Cy7 | N/A |
| NKT TCR | N/A | Mouse CD1d tetramer | NIH Tetramer Core | PE | N/A |
| CD4 | 74-12-4 | Mouse IgG2bκ | Southern Biotech | AF647 | AF647 (Invitrogen) |
| CD4 | 74-12-4 | Mouse IgG2bκ | BD Biosciences | PE | N/A |
| CD8α | 76-2-11 | Mouse IgG2aκ | Novus Biotech | AF405 | N/A |
| CD8α | 76-2-11 | Mouse IgG2aκ | Southern Biotech | AF488 | AF488 (Invitrogen) |
| CD8β | PPT23 | Mouse IgG1 | Bio-Rad | AF488 | AF488 (Invitrogen) |
| TCRδ | PGBL22A | Mouse IgG1 | WSU Mab Center | AF647 | AF647 (Invitrogen) |
| CD16 | G7 | Mouse IgG1κ | BD Biosciences | Pacific Orange | Pacific Orange (Invitrogen) |
| CD11b | M1/70 | Rat IgG2bκ | BioLegend | BV421 | N/A |
| CD14 | MIL2 | Mouse IgG2b | Bio-Rad | AF488 | AF488 (Invitrogen) |
| CD163 | 2A10/11 | Mouse IgG1 | Bio-Rad | PE | N/A |
| CD172α | 74-22-15A | Mouse IgG2bκ | BD Biosciences | PerCP | PerCP LYNX Rapid (Bio-Rad) |

CD: cluster of differentiation; Ig: immunoglobulin; PE: R-phycoerythrin; Cy: Cyanine; N/A: not applicable; NKT: natural killer T cells; TCR: T cell receptor; AF: Alexa Fluor; BV: Brilliant Violet; PerCP: Peridinin chlorophyll protein.


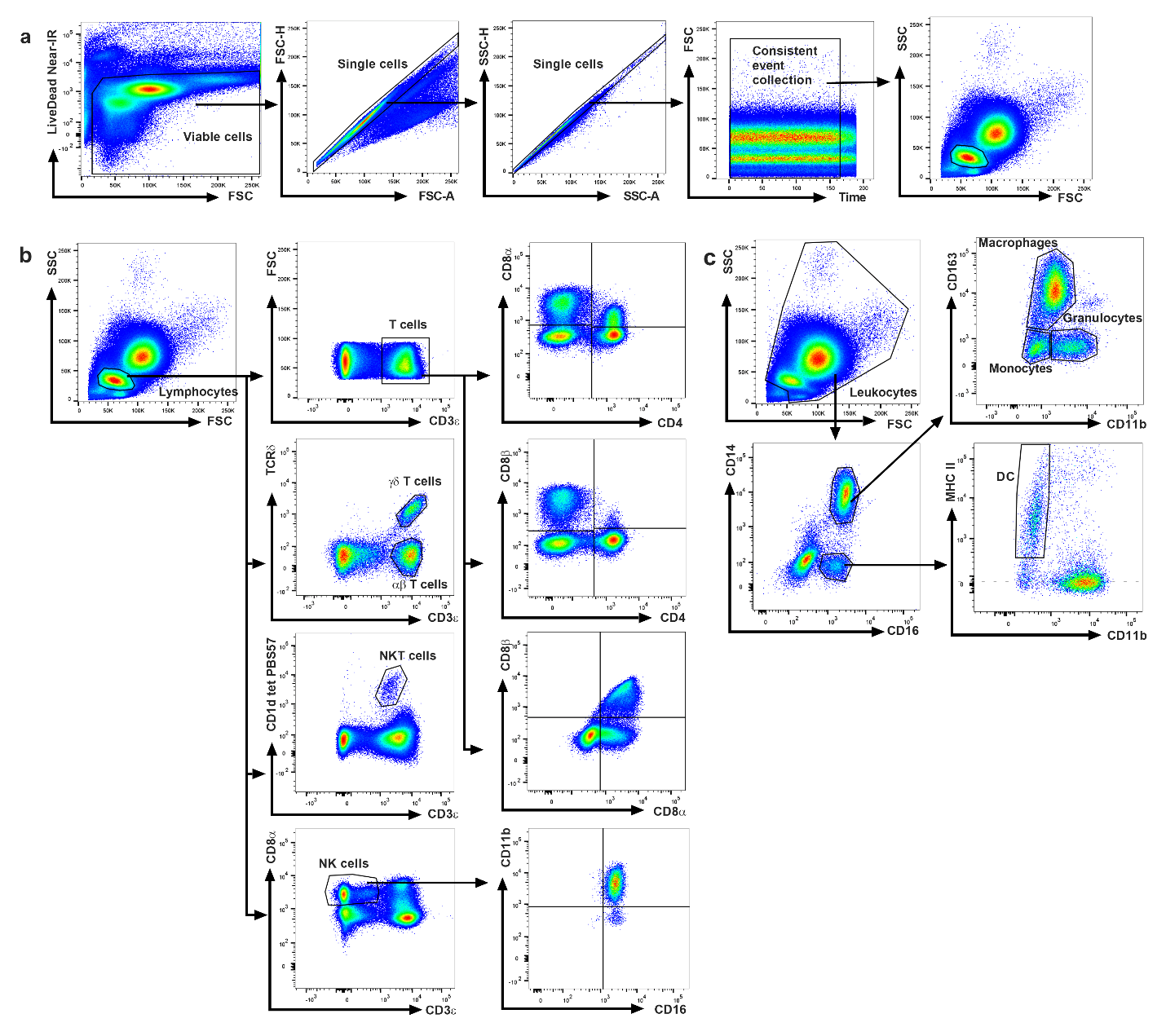


**Additional Figure 2.** Gating strategy to identify leukocyte populations in blood, bronchioalveolar lavage fluid, lung, and tracheobronchial lymph node. (a) Gating strategy to exclude dead cells, doublet cells, and cells collected during an unstable flow stream. (b) αβ and γδ T cell populations were identified by sequentially gating on the lymphocyte population and then TCRδ^+^, CD4^+^, CD8α^+^, and CD8β^+^ cells. NKT cells were identified by gating on CD3^+^ and CD1d tetramer^+^ cells. NK cells were identified by sequentially gating on the lymphocyte population and then CD8α^+^ CD3^−^ cells. Subpopulations of T and NK cells were distinguished according to CD11b and CD16 surface expression. (c) Gating strategy to distinguish polymorphonuclear cells (PMN), monocytes, macrophages, and dendritic cells. Myeloid cells were identified as CD172a^+^ cells. PMN were identified by sequentially gating on the granulocyte population and then CD14^+^ CD11b^+^ CD163^-^ cells. Monocytes were identified as CD14^+^ CD11b^-^ CD163^-^ cells. Macrophages were identified as CD14^+^ CD11b^-^ CD163^+^ cells. Dendritic cells (DC) were identified as CD14^-^ CD11b^-^ MHCII^+^ cells. FSC, forward scatter; SSC, side scatter. One representative blood sample is shown.
